# Pulmonary embolism with pulmonary hypertension or something far worse? Unveiling a rare and lethal pulmonary artery angiosarcoma: a case report

**DOI:** 10.1093/ehjcr/ytaf207

**Published:** 2025-04-25

**Authors:** Gianluca Guarnieri, Daniele Briguglia, Edoardo Conte, Daniele Andreini

**Affiliations:** Department of Biomedical and Clinical Sciences, University of Milan, via Festa del Perdono, Milan 20122, Italy; Division of University Cardiology, IRCCS Ospedale Galeazzi Sant’Ambrogio, via Belgioioso, Milan 20157, Italy; Division of University Cardiology, IRCCS Ospedale Galeazzi Sant’Ambrogio, via Belgioioso, Milan 20157, Italy; Department of Biomedical and Clinical Sciences, University of Milan, via Festa del Perdono, Milan 20122, Italy; Division of University Cardiology, IRCCS Ospedale Galeazzi Sant’Ambrogio, via Belgioioso, Milan 20157, Italy

**Keywords:** Pulmonary angiosarcoma, Pulmonary hypertension, Pulmonary embolism, Case report

## Abstract

**Background:**

Pulmonary artery angiosarcoma is a rare and highly malignant tumour. Its clinical presentation often mimics common pulmonary disorders such as pulmonary embolism or pneumonia, which frequently leads to misdiagnosis. Due to its aggressive nature and typical diagnostic delay, prognosis is generally poor, with survival often limited to a few months post-diagnosis.

**Case Summary:**

A 70-year-old female with a history of hypertension and no previous cardiac issues presented with progressive exertional dyspnoea unresponsive to anticoagulation after a diagnosis of pulmonary embolism. A chest computed tomography (CT) initially suggested a pulmonary embolism with an endoluminal filling defect in the right pulmonary artery. However, further investigation revealed a mass in the right pulmonary artery with a subsequent obliteration of the perihilar fat spaces, leading to a diagnosis of pulmonary artery angiosarcoma.

**Discussion:**

This case underscores the importance of considering pulmonary artery angiosarcoma in differential diagnoses for persistent pulmonary symptoms, especially when imaging and clinical signs are atypical for thromboembolic disease. Early recognition and a multidisciplinary approach are essential for improving outcomes in this rare but lethal malignancy, where prompt diagnosis may extend survival significantly.

**Learning points**

- Consider primary malignancy in differential diagnosis
- Malignancies arising from the heart and vessels should be suspected in patients with persistent pulmonary symptoms that do not respond to anticoagulation or when imaging findings appear atypical.
- Value of a Multidisciplinary Approach
- A timely diagnosis and optimized management of pulmonary artery angiosarcoma require collaboration among cardiologists, radiologists, and oncologists. A multidisciplinary team can more accurately assess complex cases and guide appropriate interventions, potentially improving patient outcomes.

## Background

Pulmonary artery angiosarcoma is a highly malignant and rare form of cancer that arises from the endothelial cells of the pulmonary arteries. This tumour is notoriously aggressive, often presenting with nonspecific symptoms including chest pain, coughing, and sometimes haemoptysis that mimic more common pulmonary disorders such as thromboembolism and pneumonia, which leads to frequent misdiagnoses.^1^

Due to its aggressive nature and the typical delay in diagnosis, the prognosis for patients with pulmonary artery angiosarcoma is generally poor, with survival times often not extending beyond seven months after diagnosis.^2^ Treatment options are limited but can include surgical resection, chemotherapy, and radiation therapy, though their efficacy is often restricted by the advanced stage of the disease at diagnosis.^3^ The rarity and severity of pulmonary artery angiosarcoma underscore the importance of considering it in differential diagnoses for pulmonary symptoms and utilizing advanced imaging and histopathological examination to confirm the diagnosis.

## Summary Figure

**Figure:**
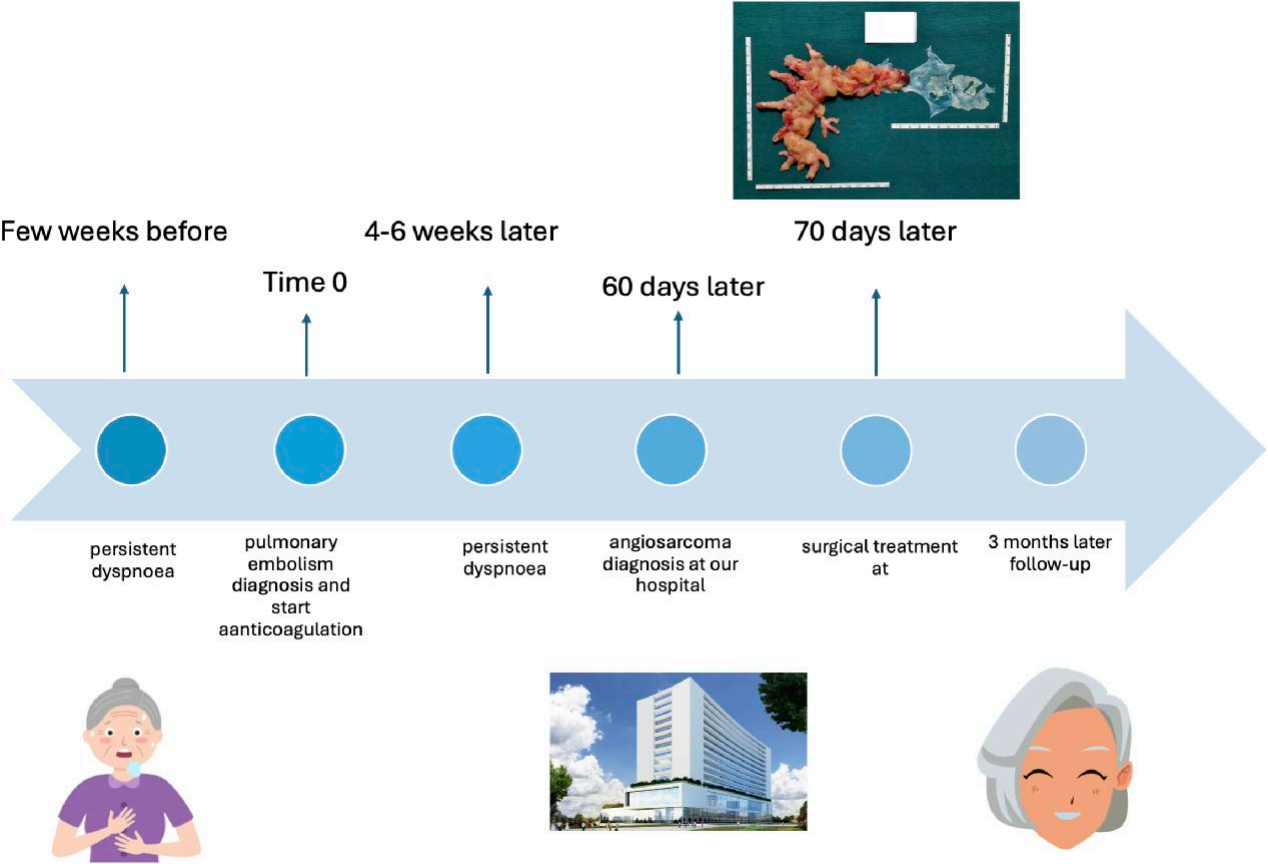


## Case description

A 70-year-old female patient presented with a history of hypertension and no previous cardiac issues. One year prior, she had undergone decompression surgery of L4-L5 and arthrodesis, and a few years earlier, she had a hysterectomy with annexectomy for a benign ovarian cyst.

A month before her current presentation, the patient was hospitalized for exertional dyspnoea that had been present for several weeks. A tomography (CT) scan revealed a significant endoluminal filling defect in the right pulmonary artery, suggestive of pulmonary thromboembolism. Echocardiography showed slightly dilated right heart chambers but a normally functioning ventricle with tricuspid annular plane systolic excursion (TAPSE) of 28 mm, fractional area change (FAC) of 40%, systolic velocity of the tricuspid annulus (S’TDI) 10 cm/s, and an indirect increase in systolic pulmonary artery pressure (sPAP, 45 mmHg). The patient was then started on therapeutic low molecular weight heparin followed by discharge with a prescription for oral anticoagulation with rivaroxaban 20 mg/day.

**Figure 1:**
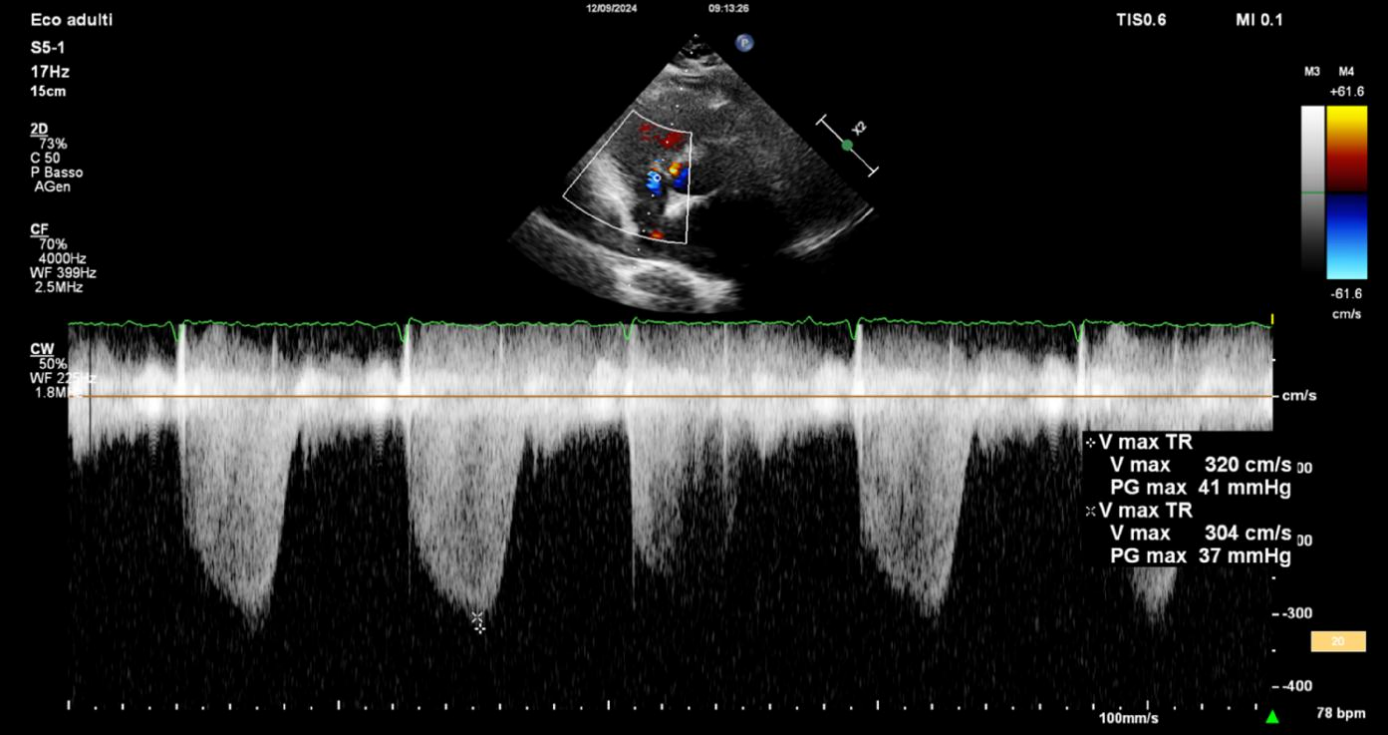
Doppler echocardiography showing a right ventricle-to-atrium pressure gradient of 37 mmHg and a regurgitation velocity greater than 2.8 m/s.

The patient visited two months later our emergency department (ED) due to a syncopal episode and reported persistent dyspnoea despite being on oral anticoagulant therapy. At the ED the patient presented with tachypnoea with minimal basal crackles on chest auscultation and in the absence of peripheral oedema. Her blood pressure was 110/70 mmHg and her heart rate was 95 bpm, with an oxygen saturation of 90%. The ECG showed sinus rhythm, normal atrio, and intra-ventricular conduction with nonspecific repolarization abnormalities. The blood tests revealed high-sensitivity troponin I (hsTnI) at 389 ng/L (reference range up to 53 ng/L) and Pro-Brain Natriuretic Peptide (proBNP) at 184 ng/mL (reference range up to 125 pg/mL); other results within normal limits. The transthoracic-echocardiogram showed normal biventricular systolic function, with a slight increase in right ventricle volumes, with systolic function (TAPSE, 25 mm; FAC, 37%) and mild tricuspid regurgitation with a velocity of 3 cm/s as shown in *Figure 1* with sPAP at 46 mmHg.

The contrast-enhanced chest CT showed an expanded filling defect in the right pulmonary artery.

**Figure 2:**
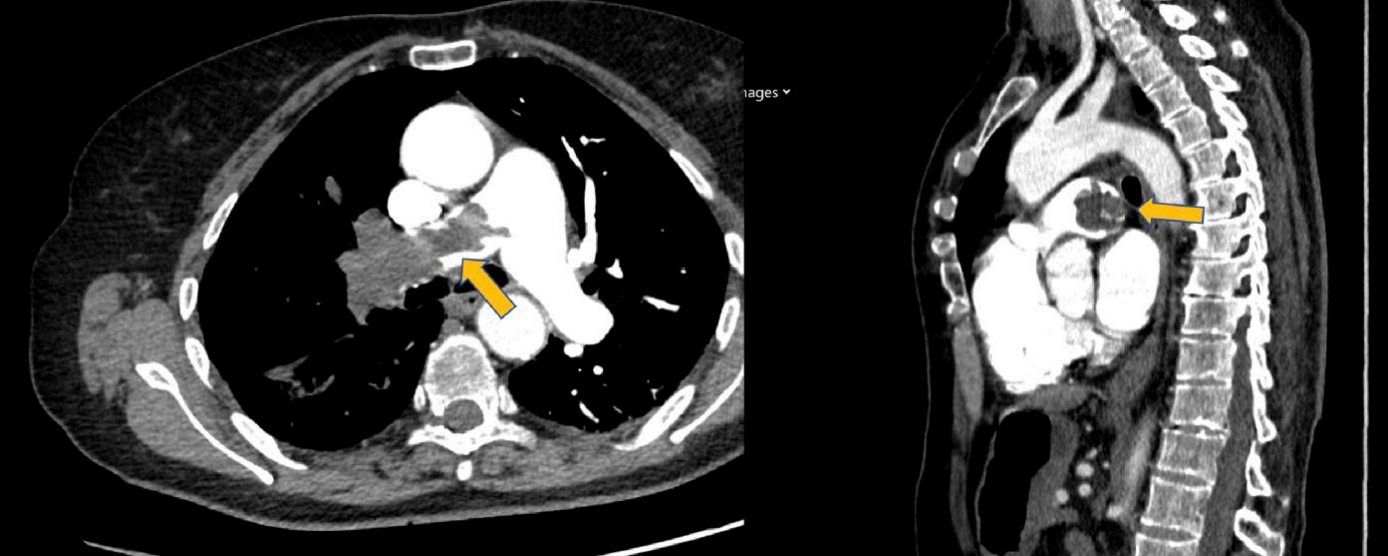
Computed tomography scan showing the pulmonary angiosarcoma, indicated with the yellow arrows.

The patient was initially admitted to the coronary care unit under the suspicion of a recurrent pulmonary embolism; however, a few hours later, the radiologist informed us that in the venous phase, there was an obliteration of the perihilar spaces, forming a mass-like appearance at this level and that these findings were suggestive of a vascular-origin neoplasia (less likely a diagnosis of pulmonary embolism), as shown in *Figure 2*.

The images were then shown to an in-house thoracic surgeon, who leaned towards a diagnosis of pulmonary embolism and recommended continuing the anticoagulant therapy. However, we decided to have the images reviewed by a second centre, known for its expertise in treating pulmonary vascular-origin neoplasms. The surgeon at this centre agreed with our radiologist’s diagnosis of pulmonary vascular-origin neoplasm. The patient also underwent a venous colour Doppler ultrasound of both the upper and lower limbs, which did not show any thrombotic formations.

**Figure 3:**
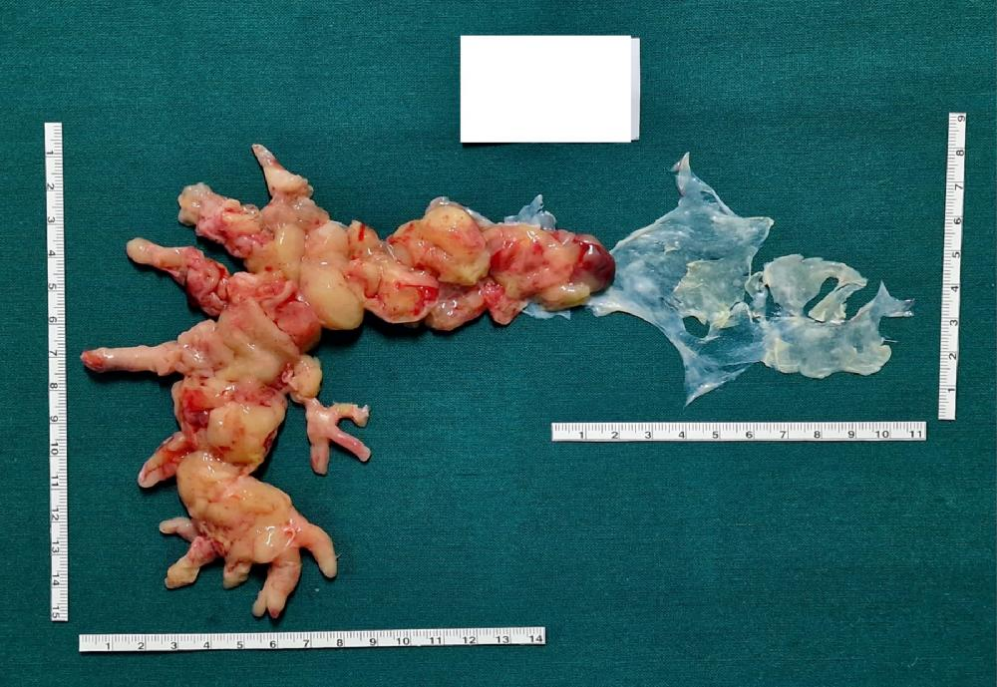
Histological examination of the resected pulmonary angiosarcoma.

The patient was subsequently transferred to this secondary level centre, where she underwent an open surgical resection of the mass, as shown in *Figure 3*. The lesion appeared macroscopically with protrusions measuring 15/14 mm. The histological examination of the resected lesion confirmed the presence of an angiosarcoma of the pulmonary artery. After some days of rehabilitation, the patient was discharged and started chemotherapy with doxorubicin administered at a standard dose of 60 mg/m² intravenously every 3 weeks, with a cumulative maximum dose of 450 mg/m, and after 3 months, is still alive.

## Discussion

Sarcomas originating from endothelial cells constitute two % of all sarcomas, which are themselves very rare tumours. The primary manifestation is dyspnoea, followed by chest pain; these patients may develop symptoms of pulmonary hypertension.^4^ Indeed, if the lesion grows over months, the patient may not only experience dyspnoea but also symptoms of right heart failure. Moreover, the patient may exhibit an increase in systolic pulmonary pressure and dilation of the right cardiac chambers, as was the case with our patient; indeed, she had been experiencing dyspnoea for several months, and two echocardiograms conducted about a month apart showed dilation of the right chambers with increased sPAP.

Diagnosis is challenging, and cases of misdiagnosis are already reported in the literature.^5^ Our patient was initially misdiagnosed at the first centre that admitted her, mistaking this neoplasm for a pulmonary embolism. Our diagnosis was facilitated by the fact that the patient remained persistently symptomatic despite anticoagulant therapy. According to literature data, the recurrence of pulmonary embolism despite anticoagulant therapy is a very rare event, affecting about two % of all venous thromboembolisms (VTEs).^6^ Moreover, embolism may coexist with malignancies arising from the heart and vessels, and anticoagulation is frequently considered in clinical practice also for this reason.

What should guide the diagnosis are both clinical and radiological characteristics; on the one hand, our patient was already on anticoagulant therapy and did not have deep vein thrombosis of distant vessels, and on the other hand, the radiological images were more suggestive of a primary form of neoplasia.

Radiologically, pulmonary artery angiosarcoma on CT imaging often presents as an intraluminal mass extending along the pulmonary artery with irregular or lobulated margins and possible infiltration of the vessel walls. These tumours can show heterogeneous enhancement following contrast administration due to their vascular nature. This contrasts with pulmonary embolism, which typically appears as filling defects confined to the vascular lumen and shows minimal or no enhancement. Additional malignant features such as local tissue invasion and metastases may also be present with angiosarcomas, unlike in cases of pulmonary embolism. The fact that the patient had this significant filling defect on only one side made the diagnosis of pulmonary embolism less likely.

This condition, in addition to being rare and challenging to diagnose, also lacks specific management guidelines. As a result, its treatment is often entrusted to highly specialized centres that rely on local expertise and experience. A multidisciplinary approach is essential, involving specialists such as cardiologists, cardiothoracic surgeons, radiologists, thoracic surgeons, and oncologists. Cardiologists play a fundamental role in the management of pulmonary angiosarcomas, particularly due to the necessity of monitoring the cardiotoxic effects associated with anthracycline-based chemotherapy. These drugs carry a significant risk of dose-dependent cardiac toxicity, making continuous cardiac monitoring essential both during and after treatment. Regular assessment of cardiac function is necessary and typically involves echocardiography, biomarker analysis (troponin, proBNP), and, when indicated, cardiac MRI. Early detection of subclinical cardiac impairment allows for the timely implementation of cardioprotective therapies, such as angiotensin-converting enzyme (ACE) inhibitors or beta-blockers, which can help reduce the risk of long-term cardiac complications.^7^

Early diagnosis is crucial; cases with survival beyond 5 years have been reported when the condition is detected in time.^3^ Thus, in instances of persistent dyspnoea, pulmonary hypertension, or recurrent pulmonary embolism unresponsive to anticoagulants with a perfusion defect in the pulmonary artery, this silent killer should always be considered.

## Conclusion

In conclusion, pulmonary artery angiosarcoma is a rare, aggressive malignancy often misdiagnosed as pulmonary embolism due to nonspecific symptoms. This case emphasizes the need to consider angiosarcoma in patients with persistent pulmonary symptoms unresponsive to standard therapies. Early diagnosis through a multidisciplinary approach can improve outcomes, highlighting the importance of awareness among clinicians.

## Data Availability

All data are incorporated into the article.

## Lead author biography

I am an intensive care cardiologist working in a coronary care unit. I completed my residency at the Centro Cardiologico Monzino and a fellowship at the Cardiocentro Ticino Institute. My main areas of interest include acute coronary syndromes, advanced heart failure, and pulmonary embolism, particularly in intermediate- and high-risk forms.

## Notes

**Consent:** Informed consent was obtained from the patient in accordance with the Committee on Publication Ethics (COPE) guidelines.
